# Human T-lymphotropic virus type I and breastfeeding; systematic review and meta-analysis of the literature

**Published:** 2018-10-07

**Authors:** Reza Boostani, Ramin Sadeghi, Amir Sabouri, Ali Ghabeli-Juibary

**Affiliations:** 1 Department of Neurology, School of Medicine, Mashhad University of Medical Sciences, Mashhad, Iran; 2 Nuclear Medicine Research Center, Mashhad University of Medical Sciences, Mashhad, Iran; 3 Department of Neurology, School of Medicine, Washington University, Saint Louis, Missouri, USA; 4 Marvdasht Shahid Motahhari Hospital, Department of Neurology, School of Medicine, Shiraz University of Medical Sciences, Shiraz, Iran

**Keywords:** Meta-Analysis, Human T-Lymphotropic Virus 1, Breast Feeding, Breast Milk, Review

## Abstract

**Background:** The human T-cell lymphotropic virus type-I (HTLV-I) is the first identified pathogenic human retrovirus. Breastfeeding has been reported to be the predominant route of vertical transmission of HTLV-I. The objective of this systematic review was to pool and evaluate the data on the transmission of HTLV-I with different infant-feeding practices on children born to HTLV-I-positive mothers. We conducted a systematic review of comparison of HTLV-I transmission risk to breastfed and bottle-fed babies.

**Methods:** We searched the following databases: MEDLINE, SID, Magiran, and Cochrane Library. The search strategy was limited to articles in English. Initial screening identified 254 citations; of these, 96 potentially relevant articles were identified. After reviewing the 96 full-text articles in detail, 7 reports met the inclusion criteria for this review.

**Results:** Pooled odds ratio (OR) and risk difference (RD) of HTLV-I transmission in the breastfed group compared to the bottle-fed infants were [OR = 3.48, 95% confidence interval (CI): 1.58-7.64, P = 0.0020, Cochran’s Q = 27.7, P = 0.0010, and I^2^ = 67.5%] and (RD = 17.1%, 95% CI: 7.5%-26.7%, P < 0.0001, Cochran’s Q = 106, P < 0.0001, and I^2^ = 91.5%). So, we have evidence to support that exclusive breast feeding more than 6 months in comparison to bottle feeding highly increases transmission rate of HTLV-I infection. We have also enough evidence to support that exclusive breast feeding up to 6 months compared to bottle feeding does not increase transmission rate of HTLV-I infection (pooled OR = 0.912, CI: 0.45-1.80; OR: 3.83, CI: 1.80-8.10, respectively).

**Conclusion:** The current meta-analysis showed that short period (less than 6 months) of breastfeeding did not increase risk of HTLV-I infection transmission from mother to child among breastfeeders and more than 6 months of breastfeeding significantly increased the risk of HTLV-I infection. However, our meta-analysis shows that refraining from breastfeeding can decrease the risk of vertical HTLV-I transmission.

## Introduction

The human T-lymphotropic virus type I (HTLV-I) is distinguished by infection of helper T-cells and is known to be the pathogenic agent of adult T-cell leukemia/lymphoma (ATLL),^1^ HTLV-I-associated myelopathy/tropical spastic paraparesis (HAM/TSP), opportunistic infections, and carcinogenesis.^2^^-^^6^

HTLV-1, which infects from 15 to 25 million individuals worldwide, is highly endemic in certain areas such as Central Africa, South-Western Japan, the Caribbean Basin, South America, Melanesia, and the Middle East.^3^^-^^7^ Retrospective and prospective epidemiological data revealed the mother-to-child transmission rate at near 20%.^4^^-^^7^

Three modes of HTLV-I transmission are known. Mother-to-child transmission is the first route, most likely due to ingestion of breast milk, breastfeeding has been reported to be the main route.^7^^,^^8^ Because of the high rate of mother-to-child transmission of HTLV-I among children breastfed for 3−6 months, since 1989 physicians recommended HTLV-I-carrier mothers to bottle feed their infants.^9^ Sexual transmission of HTLV-I infection is mainly from men to women and is the second most frequent route.^10^ Blood transfusion is the third most prevalent route, which includes HTLV-I-positive cellular units.^11^^-^^13^ Amongst these main routes, mother-to-child transmission is very important, because ATLL ensues after a lengthy incubation period since the infection about the perinatal time.^14^ Because the prognosis for patients with ATLL is extremely poor,^15^ prevention of mother-to-child transmission of HTLV-I is very important.

The first experimental evidence to support milk-borne infection of HTLV-I was the demonstration of infected cells in breast milk of carrier mothers. HTLV-I antigen was detected in milk from HTLV-I-seropositive mothers.^6^

Infected cells transferred via breast milk may be abortive for infection in the presence of enough maternal antibodies. The addition of anti-HTLV-I-containing serum to cultures of infected and normal cells inhibited HTLV-I infection.^7^

The profits of breastfeeding, like immunity against non-HTLV-I-related infectious agents, have to be balanced against the risk of HTLV-I transmission and the illness related with other feeding practices.

The optimal selection of feeding modality might be different for any individual mother and her infant by health providers about HTLV-I, and the most appropriate infant feeding choice is not determined.

This systematic review assessed the accessible evidence and data to answer this important question: whether the protective profits are associated with breastfeeding or not.

## Materials and Methods

A conceptual framework was developed to recognize transmission pathways based on route of feeding in children born to HTLV-I-positive mothers. The search terms [free text not Medical Subject Headings (MeSH) terms] were “Breastfeeding OR breast milk OR bottle feeding OR formula AND HTLV-I”. We searched the following databases: MEDLINE, SID, Magiran, and Cochrane Library. The search strategy was limited to articles in English. No date limitation was obligatory on the search and meeting abstracts were also not excluded from our search. All studies which compared the seropositivity of breastfed and bottle-fed infants born to the HTLV-I-positive mothers were included. Studies without bottle-fed control group were excluded. Reference list of the included studies were searched for any additional missed citation. 

Data were abstracted for the subsequent factors

Study characteristics: publication year, study design, follow-up duration, sample size, setting, country of study conduct, type of feeding of infants (breastfed or bottle-fed), and duration of breastfeeding.

Outcome results at the three-month intervals till the longest duration of follow-up in each study (for example HTLV-I seropositivity of children by antigen or antibody detection method) was also extracted.

A standardized data abstraction tool was developed in excels and calibrated with the team. The following data were extracted from the studies on to standardized data collection forms by the first author: characteristics of participants, study design, and study outcomes. Any disagreement was resolved by author’s discussion. The methodological quality of the included studies was appraised using the Newcastle-Ottawa Scale (NOS). 

Odds ratio (OR) and risk difference (RD) of seropositivity for HTLV-I in the breastfed infants as compared to the bottle-fed infants were used as the major effect size for meta-analysis. 

**Figure 1:**
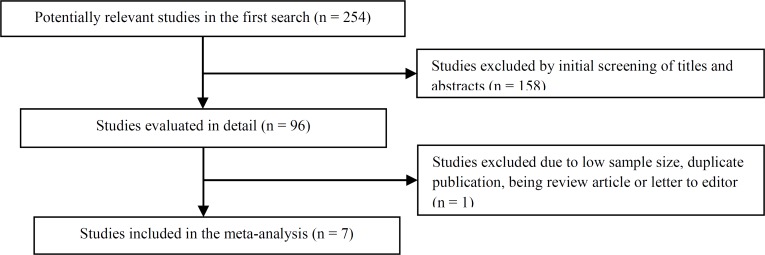
The study selection process

Random effects model was used for pooling effect size across studies. Cochran’s Q value (P < 0.0500 was considered as statistically significant) and I^2^ index were used for heterogeneity evaluation. Sub-group analysis was used for evaluating the effect of breastfeeding and follow-up duration on the HTLV-I seropositivity. 

Funnel plots, Egger’s regression intercept, and Duval-Tweedie’s trim and fill method were used for publication bias evaluation. The Funnel plot is the plot of the effect size on the x-axis and the standard errors (SEs) of the included publications on the y-axis, and asymmetry of this plot can be due to publication bias. Egger’s regression intercept is the mathematical complement of this visual assessment plot. Statistically significant results of this test indicate an outsized asymmetry in the funnel plot. All statistical analyses were done by Comprehensive Meta-Analysis (CMA) software (version 2).

## Results

***Study selection:*** Initial screening identified 254 citations and 1 additional reference was found by screening reference lists; of these, 96 potentially relevant articles were identified. After reviewing the 96 full-text articles in detail, 7 reports (observational, prospective, cohort, and retrospective controlled studies that reported outcomes relevant to this review) met the inclusion criteria for this review (Figure 1).

***Search results: ***The combined search strategy identified seven publications that met the inclusion criteria. 

***Methodological quality:*** All publications that met inclusion criteria were assessed with NOS assessment scale for their quality. 

**Figure 2:**
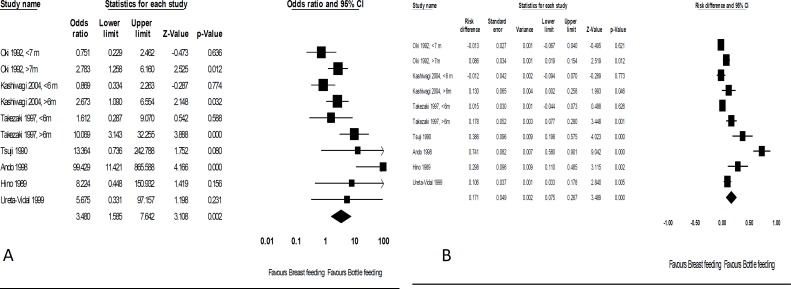
Forest plots of odds ratio (OR) (A) and risk difference (RD) (B) of human T-cell lymphotropic virus type-I (HTLV-I) transmission in the breastfed group compared to the bottled-fed infants

Figure 2 shows the forest plots of OR and RD of HTLV-I transmission in the breastfed group as compared to the bottle-fed infants. Pooled OR and RD were: (OR = 3.48, 95% CI: 1.58-7.64, P = 0.0020, Cochran’s Q = 27.7, P = 0.0010, and I^2^ = 67.5%), and (RD = 17.1%, 95% CI: 7.5%-26.7%, P < 0.0001, Cochran’s Q = 106, P < 0.0001, and I^2^ = 91.5%), respectively. 

**Figure 3:**
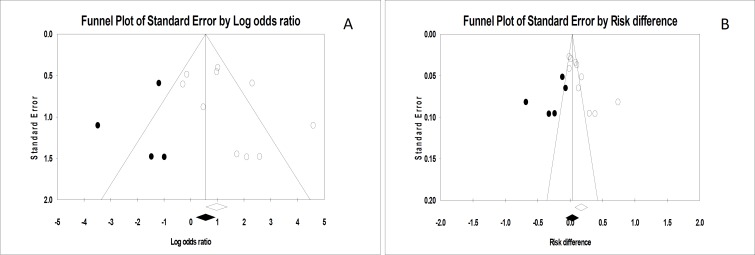
Funnel plots of odds ratio (OR) (A) and risk difference (RD) pooling (B)

Figure 3 shows the funnel plots of OR and RD pooling. Egger’s regression intercepts were 1.81 (P = 0.0900) and 6.70 (P = 0.0050), respectively. After adjustment of pooled OR and RD for possible publication bias using trim and fill method (by trimming 4 and 5 studies), the adjusted values decreased to 1.70 (95% CI: 0.72-4.00) and 3.2% (95% CI: -6.9%-13.4%), respectively.

Subgroup analyses according to duration of breastfeeding (< 6 months vs. > 6 months) showed pooled ORs of 0.91 (95% CI: 0.45-1.80) and 3.83 (95% CI: 1.80-8.10) and pooled RDs of -0.3% (95% CI: -3.8%-3.3%) and 11.9% (95% CI: 6.3%-17.5%), respectively. 

Subgroup analyses according to duration of follow up (12 months vs. 24 months) showed pooled ORs of 2.15 (95% CI: 0.15-3.00) and 15.34 (95% CI: 0.31-752.00) and pooled RDs of 15.1% (95% CI: -22.9%-53.2%) and 42.5% (95%CI: -19.4%-100%), respectively.

## Discussion

The review found that there was enough evidence to support that exclusive breastfeeding up to 6 months compared to bottle feeding did not increase transmission rate of HTLV-I infection in children born to seropositive mothers. This is probably because of gradually declining level of maternal antibody (anti-HTLV-I-IgG) in babies after birth till 6-9 months of age.^2^^,^^7^ It has been assumed that maternal HTLV-I antibody may protect children from HTLV-I infection through breast milk during the first 6 months of life.^7^ This inhibitory effect of the antibody has also been showed in an animal model.^16^ Based on our findings, shorter period of breastfeeding did not show increased risk of vertical HTLV-I transmission. 

There was sufficient evidence to support that exclusive breastfeeding more than 6 months markedly increased the transmission rate of HTLV-I infection in comparison to bottle feeding group. 

Another important factor in seroconversion after each feeding method is duration of follow up. Subgroup analysis showed that serologic status between 12 and 24 months of follow up is different and it may affect the results of our pooled studies; moreover, it has great importance in interpreting the results of our study. 

Although many risk factors for mother-to-child transmission of HTLV-I have been mentioned, like the mothers bearing a high titer of anti-HTLV-I antibody,^17^ carrying a high viral load of HTLV-I-positive cells in breast milk,^18^^-^^21^ and expressing anti-pX40 seropositivity,^22^ breastfeeding that extends after the disappearance of the maternal antibody seems to be an important risk factor for mother-to-child transmission of HTLV-I. This view is concordant with the protective role of the maternal antibody to human immunodeficiency virus 1 (HIV-1) within the first 6 months in children born to HIV-seropositive mothers.^23^^,^^24^ Another approach to decrease the risk of vertical transmission of HTLV-I is freeze-thawing of breast milk from HTLV-I-seropositive mothers. Infectivity of HTLV-I in breast milk has been lost during freezing and thawing processes. This is especially important in the countries with limited resources.^25^

 These findings suggest that length of breastfeeding should be relatively short in order to decrease the risk of HTLV-I infection among children for whom breastfeeding is necessary, especially in many developing countries all around the world. 

Other than duration of breastfeeding, mother-to-child transmission of HTLV-I is also related with older age of mother, maternal antigenemia, and high HTLV-I antibody titer level in particular to a specific immunogenic portion of gp46 envelope glycoprotein.^4^^,^^8^

Our study had some limitations. The main limitation of the systematic review was that the patient populations, follow up, and study design was not the same in all studies. We included meeting abstracts of studies in the meta-analysis, which can reduce publication bias despite this consideration, important limitation of our study is that publication bias, if present, greatly affects the results of this meta-analysis.

However, the wider applicability of these findings is unclear, in particular to areas with non-Japanese population.

## Conclusion

The current meta-analysis showed that short period (less than 6 months) of the breastfeeding did not increase chance of HTLV-I infection transmission from mother to child among breastfeeders and more than 6 months of breastfeeding highly increased transmission rate of HTLV-I infection. This study highlighted the importance of breastfeeding no longer than six months in infants born to HTLV-I-positive mothers. These estimates can serve as a basis for regulation of policy, decision making, and control measures against vertical transmission of HTLV-I.

## Notes:

**How to cite this article:** Boostani R, Sadeghi R, Sabouri A, Ghabeli-Juibary A. Human T-lymphotropic virus type I and breastfeeding; systematic review and meta-analysis of the literature. Iran J Neurol 2018; 17(4): 174-9.
